# NK Cells during Dengue Disease and Their Recognition of Dengue Virus-Infected cells

**DOI:** 10.3389/fimmu.2014.00192

**Published:** 2014-05-05

**Authors:** Davis Beltrán, Sandra López-Vergès

**Affiliations:** ^1^Department of Research in Virology and Biotechnology, Gorgas Memorial Institute for Health Studies, Panama City, Panama; ^2^Institute for Scientific Research and Technology Services (INDICASAT-AIP), Panama City, Panama; ^3^Department of Biotechnology, Acharya Nagarjuna University, Guntur, India

**Keywords:** dengue, NK cell, NK receptor, NK ligand, innate immune response

## Abstract

The innate immune response, in addition to the B- and T-cell response, plays a role in protection against dengue virus (DENV) infection and the degree of disease severity. Early activation of natural killer (NK) cells and type-I interferon-dependent immunity may be important in limiting viral replication during the early stages of DENV infection and thus reducing subsequent pathogenesis. NK cells may also produce cytokines that reduce inflammation and tissue injury. On the other hand, NK cells are also capable of inducing liver injury at early-time points of DENV infection. *In vitro*, NK cells can kill antibody-coated DENV-infected cells through antibody-dependent cell-mediated cytotoxicity. In addition, NK cells may directly recognize DENV-infected cells through their activating receptors, although the increase in HLA class I expression may allow infected cells to escape the NK response. Recently, genome-wide association studies have shown an association between *MICB* and *MICA*, which encode ligands of the activating NK receptor NKG2D, and dengue disease outcome. This review focuses on recognition of DENV-infected cells by NK cells and on the regulation of expression of NK cell ligands by DENV.

## Introduction

Dengue is a major public health problem in tropical and subtropical regions world wide and is caused by four serotypes of dengue virus (DENV-1, -2, -3, -4), a flavivirus transmitted to humans by *Aedes* mosquitoes (1). DENV infection can be asymptomatic or induce mild to severe disease, traditionally referred to as dengue hemorrhagic fever/dengue shock syndrome (DHF/DSS), which can lead to death (2). While there have been important advances in elucidating dengue pathogenesis, it is difficult to foretell if an acutely infected individual will develop the disease and to predict its severity; however, disease outcome appears to depend on the virus as well as on host genetics and prior immunity (3–5). For this reason, it is crucial to understand the immune response during DENV infection. Many studies have focused on the adaptive response, as antibodies and T cells play a crucial role in protection against infection, as well as in the pathogenesis of dengue disease (4–6). The innate immune response plays an intrinsic role at the level of the infected cell (7), but also recruits and activates innate immune cells that can eliminate the virus at early stages and induce the development of the adaptive response (8, 9). Indeed, the extent of DENV replication during the early period of infection correlates with dengue disease severity (10–14). Interstitial dendritic cells (DCs) are believed to constitute the first line of host defense against invading DENV at the anatomical sites where it replicates after the initial bite by infected mosquitoes (8). Type-I interferon-dependent immunity is known to play a critical role, and early activation of natural killer (NK) cells may also be important in limiting viral replication at the early stages of DENV infection (6, 8).

## NK Cells in Viral Infections

Natural killer cells are innate lymphocytes specialized in defense against viral and intracellular bacterial infections and tumors (15). NK cells share some characteristics with the adaptive immune system and may possess specific memory features against some viruses and antigens (16). They can be rapidly recruited into infected organs and tissues by chemoattractant factors produced by virus-infected cells and activated resident macrophages and DCs, which are a major source of the interferon IFNα/β that induces NK cell proliferation and activation (17, 18). Reciprocally, NK cells can shape DCs activation and subsequently the adaptive response (17). Once activated, NK cells fight infection by producing chemokines and anti-viral cytokines, mainly IFNγ and MIP1-β, and by recognizing and eliminating infected cells by antibody-dependent cell-mediated cytotoxicity (ADCC) or by direct recognition through their activating receptors (15). NK cells have activating and inhibitory receptors that allow them to recognize stressed cells, tumors, and pathogen-infected cells and to differentiate them from healthy cells (15, 19). Most of the inhibitory receptors recognize classic and non-classic major histocompatibility complex class I (MHC I) molecules, and many viruses decrease the expression of MHC I molecules in infected cells to escape the CD8^+^ T-cell response, thereby becoming more vulnerable to NK cell recognition (19–21).

Virus-infected cells often induce or increase the expression of ligands at their surface, allowing for recognition by NK cell activating receptors, including NKG2D, DNAM-1, CD94-NKG2C; the NCR receptors NKp46, NKp30, NKp44; and others (19, 20, 22). The ligands include host stress-induced molecules and viral proteins (20). To date, the ligands for many of the activating receptors (for example the NCRs) are still unknown, and their expression has been detected indirectly by cell staining with recombinant receptors or by blocking of killing with receptor-specific antibodies. A better characterization of NK ligands is needed. NKG2D is the most well-characterized NK activating receptor, and ligand binding leads to target killing and cytokine production (22). NKG2D ligand expression is increased by “stress” conditions, including viral infections (22, 23). In humans, eight ligands have been described for NKG2D, including MICA, MICB, and ULBP1-6. In addition to expression of NKG2D ligands on the surface of infected cells, soluble isoforms can be released into the serum, although the physiological relevance of the soluble ligands is controversial (22, 24). These soluble forms have been described in cancer patients, as well as in HIV-1-infected patients without therapy (25, 26) suggesting that soluble NKG2D ligands might be released in other viral infections.

## Role of NK Cells in Dengue Disease

Many studies suggest that NK cells play a role in the response against DENV infection, principally in the early infection stages by limiting DENV replication. A higher absolute number of NK cells was observed in patients with mild dengue fever (DF) compared with children who developed DHF (27–29). However, the percentage of NK cells and CD8^+^ T cells expressing CD69, a marker of activation, was higher early during infection of children who developed DHF (27, 30). Homchampa et al. found evidence of NK cell cytotoxicity against non-infected K562 target cells from children with acute dengue, and the cytolytic activity was increased on a per-cell basis in the early disease stages of dengue compared with healthy controls, and was even higher in the most severe form of the disease. It was suggested that this NK cell activity was associated with higher viremia in the more severe cases. Studies in adults showed that patients with mild disease had higher numbers of NK cells, with the majority of cells having increased expression of activation markers (CD69, CD38, and HLA-DR), adhesion molecules (CD11a and CD44) (Table 1), and markers of intracellular cytotoxic granules (TIA-1), in contrast to severe dengue where reduced NK cell numbers were observed (31). The authors suggest that higher NK cell percentages and activity might indicate a good prognosis of disease. In a genome-wide association study (GWAS), it was shown that the transcriptome of blood cells from children with DSS was characterized by decreased abundance of transcripts related to T and NK responses, probably not due to a difference in lymphocyte counts but to an impaired response (32). The differences observed in NK cell numbers and percentages in these studies may be explained by differences in age (children versus adults), ethnicity, time of infection, and the experimental methods to define NK cells and their activation. Nonetheless, all these observations point to the importance of NK cells and their activation during early DENV infection. The activation and phenotype of NK cells during dengue acute infection is further developed by Petitdemange et al., under the research topic “protective immune response to dengue virus infection and vaccines: perspectives from the field to the bench.”

**Table 1:** **Summary of the published findings related to ligands for receptors on NK cells and dengue virus infection**.

Ligand	Study type	Observation	Receptor on NK cells	Probable effect	Reference
ICAM-1	Acute patients’ lymphocytes	Adhesion molecules (ICAM-1 and LFA-1) are increased in NK cells in the acute phase of dengue disease	LFA-1	adhesion	Azeredo et al. (31)
DENV-specific antibodies	*In vitro* with activated peripheral blood lymphocytes	NK cells are the principal cells active in the ADCC against DENV-infected cells	CD16	Activation, ADCC	Kurane et al. (42, 43) and Laoprasopwattana et al. (44)
DENV ligands (probably: protein E)	*In vitro* activation of NK cell toward E protein and VLP of flaviviruses	Protein–protein interaction between rNKp44 and cells expressing DENV-E proteins. Interaction of NKp44 with E from another flavivirus (WNV) induces killing and IFNγ production	NKp44	Activation?	Hershkovitz et al. (55)
MICA	Allele association	MICA alleles associated with dengue symptomatic infection	NKG2D	Activation?	García et al. (59)
MICB	Allele association, GWAS	MICB alleles associated with symptomatic infection and dengue shock syndrome	NKG2D	Activation?	García et al. (59), Khor et al. (60), Whitehorn et al. (61)

The protective role of NK cells in the response against DENV is supported in mice models of the disease. In immunocompetent A/J mice, the early activation of NK and B cells was associated with the control of the viral load and the prevention of disease (33, 34). In C57BL/6 mice, the recruitment of NK and NKT cells by mast cells to the site of infection was also crucial for viral clearance (35), underlining the importance of these cells during the host early response against DENV. The early recruitment of NK cells to the liver was induced in part by CXCL10 (IP-10), and in the liver NK cells produce effector molecules (perforin, granzyme A, and granzyme B) needed for viral clearance (36). On the other hand, NK cells appear to play not only a protective role in dengue disease, as it was shown that after intrahepatic infiltration, NK cells were responsible for cell death in the liver at the early phase of infection, whereas CD8^+^ T cells were responsible for damage later (37). The mechanism of this cell death has not been elucidated, but the authors suggested that NK cells were killing DENV-infected cells. It is possible that under some conditions, the elimination of DENV-infected cells by NK cells can be exacerbated in a way that the immune effector cells become responsible for organ injury. Similarly, NKT cells in some conditions can be detrimental during dengue pathogenesis, in part by inducing NK cells and neutrophils activation (38).

Natural killer cells may play an important role in early DENV infection *in vivo* also by producing together with γδ T cells, IL-22 and IL-17A, which may influence dengue disease outcome (39). NK, NKT, and T cells that can respond in a non-TcR-dependent fashion are a major source of IFNγ in immune responses induced by inactivated DENV (40); however, in this context, the frequency of IFNγ-producing cells observed was very low. Using C57BL/6 mice, a study confirmed the role of NK cells in the early IFNγ production induced by IL-12 and IL-18 in response to DENV, key for the control of viral load and DENV-2-associated disease severity and lethality (41).

Natural killer cells can mediate ADCC against DENV-infected cells (42, 43) and this mechanism may be important during secondary infections when antibodies to DENV are present. Indeed, ADCC activity in plasma obtained before secondary DENV-2 or DENV-3 infection correlated with serotype-specific neutralizing antibody titers, anti-DENV IgG1 levels, and a multitypic PRNT_50_ pattern (44). Interestingly, a higher level of ADCC activity measured before secondary DENV-3 infection was associated with lower subsequent viremia, which suggests a protective role for antibodies and NK cells; however, this association was not observed for secondary DENV-2 infection. In another study, ADCC was correlated with DENV surface antigen expression, suggesting recognition by anti-DENV antibodies (42), whose Fc region is then recognized by the activating low affinity CD16 (FcγRIII) receptor on NK cells (45).

Human NK cells (CD3^−^CD16^+^CD11b^+^ cells) can lyse DENV-infected cells to a greater level than uninfected cells even in the absence of antibodies, suggesting a mechanism of direct recognition as well (42, 43). These studies were performed using DENV-infected Raji cells as targets. The NK receptors and their ligands implicated in the direct recognition of DENV-infected cells have not been fully elucidated, indicating the need for future studies.

## Expression of NK Cell Ligands during Dengue Disease

Viruses try to escape the immune response of the host. As NK cells are crucial players in the anti-viral response, many viruses induce the up-regulation of MHC I, which serve as ligands for NK inhibitory receptors, in order to dampen the NK cell response even if enhancing expression of MHC I might increase their recognition by CD8^+^ T cells (20). DENV and other flaviviruses induce the up-regulation of MHC I (46–51). Expression of flavivirus pr-M protein in hamster cells induced increased surface expression of MHC I, although the authors suggested this could be an incidental consequence of viral assembly rather than a specific mechanism of immune evasion (52). It has also been shown that human cell lines expressing the non-structural (NS) proteins of DENV up-regulate MHC I (HLA-A, -B, -C) expression at their surface by TAP-dependent and TAP-independent pathways (46). This resulted in a lower sensibility to lysis by NK cells, probably due to recognition by the corresponding inhibitory receptors (e.g., KIR2DL1 for HLA-C) and the inhibitory receptor LIR-1 that recognizes all MHC I proteins (Table 1). Further studies are needed to determine if MHC I up-regulation by DENV is the response to a particular viral protein, viral replication itself, or type-I IFNs induced by viral infection, and whether it plays a role in immune evasion of NK cells *in vivo*. Neurotropic flaviviruses, such as West Nile virus (WNV), can transiently activate and then suppress NK cell activity (53). Future studies are needed to determine if this happens during DENV infection by analyzing NK cell activity at different time points post-infection.

Even if DENV NS proteins induce MHC I up-regulation, NK cells can kill DENV-infected cells (28, 42, 43), suggesting that the signals for NK cell activation overcome the inhibitory signals. As described above, NK cells can kill DENV-infected cells by ADCC mediated by CD16 (42, 44) (Table 1), which is expressed on resting human NK cells and can induce a strong activating signal leading to cytolysis (20, 54). Interestingly, NK cells can also kill DENV-infected cells in the absence of antibodies (42), implicating a direct recognition by NK cell activating receptors. It has been reported that the activating receptor NKp44 can interact with the envelope protein (E) of DENV (55) (Table 1). NKp44 has also been implicated in killing WNV-infected cells after blocking the inhibitory receptor LIR-1 on NK cells, and this also induced IFNγ production. Further studies are necessary to determine if recognition of DENV-E by NKp44 on NK cells can induce a similar response against DENV-infected cells. NKp44 is expressed on activated, but not resting, NK cells (56) and can trigger NK cell killing of both tumor and virus-infected cells (57, 58).

**Figure 1:**
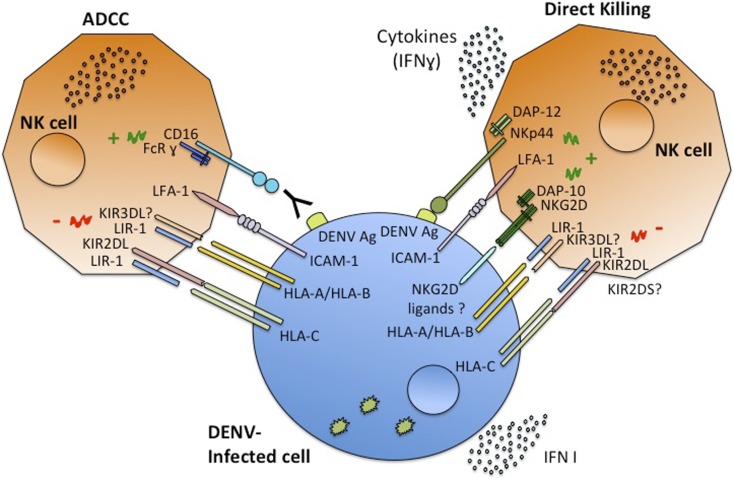
**Schematic model of NK cells interaction with DENV-infected cells**. NK cells recognize infected cells through the interaction of their receptors with ligands on the infected cells, the outcome depends on the balance between activating or inhibitory signals. Once activated NK cells are capable of ADCC or direct cytotoxicity, and of producing cytokines, mainly IFNγ.

Recently, a sequencing-based typing method and genotyping of asymptomatic DF and DHF patients in Cuba uncovered an association of certain alleles of the *MICA* and *MICB* genes (MICA*008 and MICB*008) with symptomatic DENV infection (59) (Table 1). The importance of MICB in dengue susceptibility was also indicated by GWAS with a large number of pediatric cases in Vietnam, where certain *MICB* and *PLCE1* alleles showed a significant association with DSS (60). These results were confirmed by a study showing that *MICB* rs3132468 and *PLCE1* risk genotypes were also associated with less severe clinical phenotypes of dengue in adults as well as with DENV infection in infants (61). This strongly suggests a role for this *MICB* variant in susceptibility to overall clinically apparent dengue disease. It still has not been determined whether the association between these NKG2D ligands and dengue clinical responses is directly due to the function of MICA/B molecules in dengue pathogenesis. Nonetheless, the importance of NKG2D ligands in the NK cell response against other viral infections (22) supports this hypothesis. Given the role of MICB in activation of NK, NKT, and CD8^+^ T cells through the NKG2D receptor, these findings support a central role for these cell types in shaping the outcome of DENV infection. It is plausible that the *MICB* risk-associated phenotype is associated with an impaired NK cell response, potentially resulting in a higher *in vivo* virus titer and an increased risk of developing both symptomatic and severe dengue. Furthermore, inefficient induction of cytokines secreted by NK cells might result in dysregulated T-cell responses that may also shape the clinical phenotype (62). NKG2D ligand (MICA/B) expression on DENV-infected cells may allow direct recognition by NK cells, which might be important for the early innate immune response against DENV infection, leading to either more effective control of viral infection or alternatively contributing to the disease pathology. The expression pattern of these NKG2D ligands in DENV-infected cells *in vitro* and *in vivo* needs to be determined. It is also necessary to determine if in acute viral infections such as DENV, the production of soluble NKG2D ligands can also be observed and if this impacts dengue clinical manifestations. Because ligands of other NK activating receptors, such as DNAM-1, can be induced by “stress” (23), further studies are needed to characterize all the NK receptor ligands induced during DENV infection, and the results may depend on the cell-type analyzed, as well as the DENV serotype. A complete characterization of DENV-infected cell recognition by NK cells (Figure 1) is crucial to better understanding the role of these cells in dengue disease.

## Conclusion

The innate immune response, and particularly type-I IFN and NK cells, plays a key role during the early infection events due to its ability to rapidly limit viral dissemination and to affect the antigen-specific, adaptive immune responses to effectively clear pathogens (8, 9). More studies in animal models and human populations will enable deciphering NK cell responses during DENV infection *in vivo*. Furthermore, *in vitro* experiments will also be needed to determine which NK receptors and ligands are implicated in DENV-infected cell recognition and NK cell-mediated killing and cytokine production. To date, only the up-regulation of MHC I and the induction of a putative NKp44 ligand (DENV-E protein) have been reported in DENV-infected cells. Are there other ligands that play a role in NK cell recognition induced during DENV infection? Genetic studies suggest an important role for the NKG2D ligands MICA and MICB; however, as yet no functional experiments have validated this hypothesis. There is still much to do to determine if ligands of other NK receptors, for example HLA-E for CD94/NKG2A-C and the ligands for the activating receptors 2B4 and DNAM-1, are induced or repressed during DENV infection, and whether this has an implication in the immune response to DENV and its clinical outcome. Finally, the characterization of NK receptor ligands and the NK cell phenotype in patients’ blood cells will provide insights into the NK cell subsets activated during dengue disease. It will also establish possible associations between NK cell activity or NK cell ligand expression and protection from disease and/or increased dengue severity. Whether DENV interferes with innate anti-viral immunity mediated by NK cells at early times of infection and whether DENV virulence might be associated with its ability to counter the host cell defenses are critical issues that remain to be elucidated.

## Conflict of Interest Statement

The authors declare that the research was conducted in the absence of any commercial or financial relationships that could be construed as a potential conflict of interest.

## References

[1] Halstead SB Dengue. Lancet (2007) 370(9599):1644–52 10.1016/S0140-6736(07)61687-0 17993365

[2] Anon. Dengue: Guidelines for Diagnosis, Treatment, Prevention and Control. Geneva: World Health Organization and the Special Programme for Research and Training in Tropical diseases (2009). p. 25–8

[3] OhAinle M Balmaseda A Macalalad AR Tellez Y Zody MC Saborío S Dynamics of dengue disease severity determined by the interplay between viral genetics and serotype-specific immunity. Sci Transl Med (2011) 3(114):114ra128 10.1126/scitranslmed.3003084 22190239 PMC4517192

[4] Whitehorn J Simmons CP The pathogenesis of dengue. Vaccine (2011) 29(42):7221–8 10.1016/j.vaccine.2011.07.022 21781999

[5] Yacoub S Mongkolsapaya J Screaton G The pathogenesis of dengue. Curr Opin Infect Dis (2013) 26(3):284–9 10.1097/QCO.0b013e32835fb938 23449140

[6] Kurane I Innis BL Nimmannitya S Nisalak A Rothman AL Livingston PG Human immune response to dengue virus. Southeast Asian J Trop Med Public Health (1990) 21(4):658–62 1983049

[7] Green AM Beatty PR Hadjilaou A Harris E Innate immunity to dengue virus infection and subversion of antiviral responses. J Mol Biol (2014) 426(6):1148–60 10.1016/j.jmb.2013.11.023 24316047 PMC4174300

[8] Navarro-Sánchez E Desprès P Cedillo-Barrón L Innate immune responses to dengue virus. Arch Med Res (2005) 36(5):425–35 10.1038/jid.2012.76 16099317

[9] Sun P Kochel TJ The battle between infection and host immune responses of dengue virus and its implication in dengue disease pathogenesis. ScientificWorldJournal (2013) 2013:843469 10.1155/2013/843469 23476150 PMC3582169

[10] Durbin AP Vargas MJ Wanionek K Hammond SN Gordon A Rocha C Phenotyping of peripheral blood mononuclear cells during acute dengue illness demonstrates infection and increased activation of monocytes in severe cases compared to classic dengue fever. Virology (2008) 376(2):429–35 10.1016/j.virol.2008.03.028 18452966 PMC2546568

[11] Duyen HT Ngoc TV Hado T Hang VT Kieu NT Young PR Kinetics of plasma viremia and soluble nonstructural protein 1 concentrations in dengue: differential effects according to serotype and immune status. J Infect Dis (2011) 203(9):1292–300 10.1093/infdis/jir014 21335562 PMC3069728

[12] Libraty DH Young PR Pickering D Endy TP Kalayanarooj S Green S High circulating levels of the dengue virus nonstructural protein NS1 early in dengue illness correlate with the development of dengue hemorrhagic fever. J Infect Dis (2002) 186(8):1165–8 10.1086/343813 12355369

[13] Srikiatkhachorn A Wichit S Gibbons RV Green S Libraty DH Endy TP Dengue viral RNA levels in peripheral blood mononuclear cells are associated with disease severity and preexisting dengue immune status. PLoS One (2012) 7(12):e51335 10.1371/journal.pone.0051335 23284680 PMC3526575

[14] Tricou V Minh NN Farrar J Tran HT Simmons CP Kinetics of viremia and NS1 antigenemia are shaped by immune status and virus serotype in adults with dengue. PLoS Negl Trop Dis (2011) 5(9):e1309 10.1371/journal.pntd.0001309 21909448 PMC3167785

[15] Sun JC Lanier LL NK cell development, homeostasis and function: parallels with CD8^+^ T cells. Nat Rev Immunol (2011) 11(10):645–57 10.1038/nri3044 21869816 PMC4408539

[16] Min-Oo G Kamimura Y Hendricks DW Nabekura T Lanier LL Natural killer cells: walking three paths down memory lane. Trends Immunol (2013) 34(6):251–8 10.1016/j.it.2013.02.005 23499559 PMC3674190

[17] Della Chiesa M Sivori S Castriconi R Marcenaro E Moretta A Pathogen-induced private conversations between natural killer and dendritic cells. Trends Microbiol (2005) 13(3):128–36 10.1016/j.tim.2005.01.006 15737731

[18] Robertson MJ Role of chemokines in the biology of natural killer cells. J Leukoc Biol (2002) 71(2):173–83 11818437

[19] Finton KA Strong RK Structural insights into activation of antiviral NK cell responses. Immunol Rev (2012) 250(1):239–57 10.1111/j.1600-065X.2012.01168.x 23046134 PMC3471384

[20] Lanier LL Evolutionary struggles between NK cells and viruses. Nat Rev Immunol (2008) 8(4):259–68 10.1038/nri2276 18340344 PMC2584366

[21] López-Botet M Angulo A Gumá M Natural killer cell receptors for major histocompatibility complex class I and related molecules in cytomegalovirus infection. Tissue Antigens (2004) 63(3):195–203 10.1111/j.1399-0039.2004.00210.x 14989708

[22] Champsaur M Lanier LL Effect of NKG2D ligand expression on host immune responses. Immunol Rev (2010) 235(1):267–85 10.1111/j.0105-2896.2010.00893.x 20536569 PMC2885032

[23] Cerboni C Fionda C Soriani A Zingoni A Doria M Cippitelli M The DNA damage response: a common pathway in the regulation of NKG2D and DNAM-1 ligand expression in normal, infected, and cancer cells. Front Immunol (2014) 4:508 10.3389/fimmu.2013.00508 24432022 PMC3882864

[24] Groh V Wu J Yee C Spies T Tumour-derived soluble MIC ligands impair expression of NKG2D and T-cell activation. Nature (2002) 419(6908):734–8 10.1038/nature01112 12384702

[25] Matusali G Tchidjou HK Pontrelli G Bernardi S D’Ettorre G Vullo V Soluble ligands for the NKG2D receptor are released during HIV-1 infection and impair NKG2D expression and cytotoxicity of NK cells. FASEB J (2013) 27(6):2440–50 10.1096/fj.12-223057 23395909

[26] Nolting A Dugast AS Rihn S Luteijn R Carrington MF Kane K MHC class I chain-related protein A shedding in chronic HIV-1 infection is associated with profound NK cell dysfunction. Virology (2010) 406(1):12–20 10.1016/j.virol.2010.05.014 20667578 PMC2932841

[27] Green S Pichyangkul S Vaughn DW Kalayanarooj S Nimmannitya S Nisalak A Early CD69 expression on peripheral blood lymphocytes from children with dengue hemorrhagic fever. J Infect Dis (1999) 180(5):1429–35 10.1016/j.jmb.2013.11.023 10515800

[28] Homchampa P Sarasombath S Suvatte V Vongskul M Natural killer cells in dengue hemorrhagic fever/dengue shock syndrome. Asian Pac J Allergy Immunol (1988) 6(2):95–102 3064759

[29] Wahid SF Sanusi S Zawawi MM Ali RA A comparison of the pattern of liver involvement in dengue hemorrhagic fever with classic dengue fever. Southeast Asian J Trop Med Public Health (2000) 31(2):259–63 11127322

[30] Chau TNB Quyen NT Thuy TT Tuan NM Hoang DM Dung NT Dengue in Vietnamese infants – results of infection-enhancement assays correlate with age-related disease epidemiology, and cellular immune responses correlate with disease severity. J Infect Dis (2008) 198(4):516–24 10.1086/590117 18598189 PMC2730540

[31] Azeredo EL De Oliveira-Pinto LM Zagne SM Cerqueira DI Nogueira RM Kubelka CF NK cells, displaying early activation, cytotoxicity and adhesion molecules, are associated with mild dengue disease. Clin Exp Immunol (2006) 143(2):345–56 10.1111/j.1365-2249.2006.02996.x 16412060 PMC1809585

[32] Devignot S Sapet C Duong V Bergon A Rihet P Ong S Genome-wide expression profiling deciphers host responses altered during dengue shock syndrome and reveals the role of innate immunity in severe dengue. PLoS One (2010) 5(7):e11671 10.1371/journal.pone.0011671 20652028 PMC2907396

[33] Shresta S Kyle JL Robert Beatty P Harris E Early activation of natural killer and B cells in response to primary dengue virus infection in A/J mice. Virology (2004) 319(2):262–73 10.1016/j.virol.2003.09.048 14980486

[34] Shresta S Sharar KL Prigozhin DM Snider HM Beatty PR Harris E Critical roles for both STAT1-dependent and STAT1-independent pathways in the control of primary dengue virus infection in mice. J Immunol (2005) 175(6):3946–54 10.4049/jimmunol.175.6.3946 16148142

[35] St John AL Rathore AP Yap H Ng ML Metcalfe DD Vasudevan SG Immune surveillance by mast cells during dengue infection promotes natural killer (NK) and NKT-cell recruitment and viral clearance. Proc Natl Acad Sci USA (2011) 108(22):9190–5 10.1073/pnas.1105079108 21576486 PMC3107258

[36] Chen J-P Lu HL Lai SL Campanella GS Sung JM Lu MY Dengue virus induces expression of CXC chemokine ligand 10/IFN-gamma-inducible protein 10, which competitively inhibits viral binding to cell surface heparan sulfate. J Immunol (2006) 177(5):3185–92 10.4049/jimmunol.177.5.3185 16920957

[37] Sung JM Lee C-K Wu-Hsieh BA Intrahepatic infiltrating NK and CD8 T cells cause liver cell death in different phases of dengue virus infection. PLoS One (2012) 7(9):e46292 10.1371/journal.pone.0046292 23050007 PMC3458800

[38] Renneson J Guabiraba R Maillet I Marques RE Ivanov S Fontaine J A detrimental role for invariant natural killer T cells in the pathogenesis of experimental dengue virus infection. Am J Pathol (2011) 179(4):1872–83 10.1016/j.ajpath.2011.06.023 21843496 PMC3181339

[39] Guabiraba R Besnard AG Marques RE Maillet I Fagundes CT Conceição TM IL-22 modulates IL-17A production and controls inflammation and tissue damage in experimental dengue infection. Eur J Immunol (2013) 43(6):1529–44 10.1002/eji.201243229 23505056

[40] Suwannasaen D Romphruk A Leelayuwat C Lertmemongkolchai G Bystander T cells in human immune responses to dengue antigens. BMC Immunol (2010) 11:47 10.1186/1471-2172-11-47 20854672 PMC2949776

[41] Fagundes CT Costa VV Cisalpino D Amaral FA Souza PR Souza RS IFN-γ production depends on IL-12 and IL-18 combined action and mediates host resistance to dengue virus infection in a nitric oxide-dependent manner. PLoS Negl Trop Dis (2011) 5(12):e1449 10.1371/journal.pntd.0001449 22206036 PMC3243710

[42] Kurane I Hebblewaite D Brandt WE Ennis FA Lysis of dengue virus-infected cells by natural cell-mediated cytotoxicity and antibody-dependent cell-mediated cytotoxicity. J Virol (1984) 52(1):223–30 6207308 10.1128/jvi.52.1.223-230.1984 PMC254509

[43] Kurane I Hebblewaite D Ennis FA Characterization with monoclonal antibodies of human lymphocytes active in natural killing and antibody-dependent cell-mediated cytotoxicity of dengue virus-infected cells. Immunology (1986) 58(3):429–36 3089915 PMC1453483

[44] Laoprasopwattana K Libraty DH Endy TP Nisalak A Chunsuttiwat S Ennis FA Antibody-dependent cellular cytotoxicity mediated by plasma obtained before secondary dengue virus infections: potential involvement in early control of viral replication. J Infect Dis (2007) 195(8):1108–16 10.1086/512860 17357046

[45] Lanier LL Kipps TJ Phillips JH Functional properties of a unique subset of cytotoxic CD3+ T lymphocytes that express Fc receptors for IgG (CD16/Leu-11 antigen). J Exp Med (1985) 162(6):2089–106 10.1084/jem.162.6.2089 2415663 PMC2187997

[46] Hershkovitz O Zilka A Bar-Ilan A Abutbul S Davidson A Mazzon M Dengue virus replicon expressing the nonstructural proteins suffices to enhance membrane expression of HLA class I and inhibit lysis by human NK cells. J Virol (2008) 82(15):7666–76 10.1128/JVI.02274-07 18508882 PMC2493327

[47] Lobigs M Blanden RV Müllbacher A Flavivirus-induced up-regulation of MHC class I antigens; implications for the induction of CD8+ T-cell-mediated autoimmunity. Immunol Rev (1996) 152:5–19 10.1111/j.1600-065X.1996.tb00908.x 8930665 PMC7165549

[48] Lobigs M Müllbacher A Regner M MHC class I up-regulation by flaviviruses: immune interaction with unknown advantage to host or pathogen. Immunol Cell Biol (2003) 81(3):217–23 10.1046/j.1440-1711.2003.01161.x 12752686

[49] Momburg F Müllbacher A Lobigs M Modulation of transporter associated with antigen processing (tap) -mediated peptide import into the endoplasmic reticulum by flavivirus infection. J Virol (2001) 75(12):5663–71 10.1128/JVI.75.12.5663-5671.2001 11356974 PMC114279

[50] Müllbacher A Lobigs M Up-regulation of MHC class I by flavivirus-induced peptide translocation into the endoplasmic reticulum. Immunity (1995) 3(2):207–14 10.1016/1074-7613(95)90090-X 7544229

[51] Ye J Zhu B Fu ZF Chen H Cao S Immune evasion strategies of flaviviruses. Vaccine (2013) 31(3):461–71 10.1016/j.vaccine.2012.11.015 23153447

[52] Lobigs M Müllbacher A Lee E Evidence that a mechanism for efficient flavivirus budding upregulates MHC class I. Immunol Cell Biol (2004) 82(2):184–8 10.1046/j.0818-9641.2004.01218.x 15061772

[53] Vargin VV Semenov BF Changes of natural killer cell activity in different mouse lines by acute and asymptomatic flavivirus infections. Acta Virol (1986) 30(4):303–8 2876611

[54] Bryceson YT March ME Ljunggren H-G Long EO Synergy among receptors on resting NK cells for the activation of natural cytotoxicity and cytokine secretion. Blood (2006) 107(1):159–66 10.1182/blood-2005-04-1351 16150947 PMC1895346

[55] Hershkovitz O Rosental B Rosenberg LA Navarro-Sanchez ME Jivov S Zilka A NKp44 receptor mediates interaction of the envelope glycoproteins from the West Nile and dengue viruses with NK cells. J Immunol (2009) 183(4):2610–21 10.4049/jimmunol.0802806 19635919 PMC2768489

[56] Vitale M Bottino C Sivori S Sanseverino L Castriconi R Marcenaro E NKp44, a novel triggering surface molecule specifically expressed by activated natural killer cells, is involved in non-major histocompatibility complex-restricted tumor cell lysis. J Exp Med (1998) 187(12):2065–72 10.1084/jem.187.12.2065 9625766 PMC2212362

[57] Arnon TI Markel G Mandelboim O Tumor and viral recognition by natural killer cells receptors. Semin Cancer Biol (2006) 16(5):348–58 10.1016/j.semcancer.2006.07.005 16893656

[58] Moretta A Bottino C Vitale M Pende D Cantoni C Mingari MC Activating receptors and coreceptors involved in human natural killer cell-mediated cytolysis. Annu Rev Immunol (2001) 19:197–223 10.1146/annurev.immunol.19.1.197 11244035

[59] García G del Puerto F Pérez AB Sierra B Aguirre E Kikuchi M Association of MICA and MICB alleles with symptomatic dengue infection. Hum Immunol (2011) 72(10):904–7 10.1016/j.humimm.2011.06.010 21762746

[60] Khor CC Chau TN Pang J Davila S Long HT Ong RT Genome-wide association study identifies susceptibility loci for dengue shock syndrome at MICB and PLCE1. Nat Genet (2011) 43(11):1139–41 10.1038/ng.960 22001756 PMC3223402

[61] Whitehorn J Chau TN Nguyet NM Kien DT Quyen NT Trung DT Genetic variants of MICB and PLCE1 and associations with non-severe dengue. PLoS One (2013) 8(3):e59067 10.1371/journal.pone.0059067 23536857 PMC3594159

[62] Lang PA Lang KS Xu HC Grusdat M Parish IA Recher M Natural killer cell activation enhances immune pathology and promotes chronic infection by limiting CD8+ T-cell immunity. Proc Natl Acad Sci U S A (2012) 109(4):1210–5 10.1073/pnas.1118834109 22167808 PMC3268324

